# Structural Analysis on the Severe Acute Respiratory Syndrome Coronavirus 2 Non-structural Protein 13 Mutants Revealed Altered Bonding Network With TANK Binding Kinase 1 to Evade Host Immune System

**DOI:** 10.3389/fmicb.2021.789062

**Published:** 2021-12-01

**Authors:** Farooq Rashid, Muhammad Suleman, Abdullah Shah, Emmanuel Enoch Dzakah, Shuyi Chen, Haiying Wang, Shixing Tang

**Affiliations:** ^1^Dermatology Hospital, Southern Medical University, Guangzhou, China; ^2^Centre for Biotechnology and Microbiology, University of Swat, Mingora, Pakistan; ^3^Department of Biotechnology, Shaheed Benazir Bhutto University, Sheringal, Dir, Pakistan; ^4^Department of Molecular Biology and Biotechnology, School of Biological Sciences, College of Agriculture and Natural Sciences, University of Cape Coast, Cape Coast, Ghana; ^5^Guangdong Provincial Key Laboratory of Tropical Disease Research, School of Public Health, Southern Medical University, Guangzhou, China; ^6^Wenzhou Institute, University of Chinese Academy of Sciences, Wenzhou, China

**Keywords:** SARS-CoV-2, NSP13 mutants, TBK1, protein-protein docking, MD simulations

## Abstract

Mutations in severe acute respiratory syndrome coronavirus 2 (SARS-CoV-2) have made this virus more infectious. Previous studies have confirmed that non-structural protein 13 (NSP13) plays an important role in immune evasion by physically interacting with TANK binding kinase 1 (TBK1) to inhibit IFNβ production. Mutations have been reported in NSP13; hence, in the current study, biophysical and structural modeling methodologies were adapted to dissect the influence of major mutations in NSP13, i.e., P77L, Q88H, D260Y, E341D, and M429I, on its binding to the TBK1 and to escape the human immune system. The results revealed that these mutations significantly affected the binding of NSP13 and TBK1 by altering the hydrogen bonding network and dynamic structural features. The stability, flexibility, and compactness of these mutants displayed different dynamic features, which are the basis for immune evasion. Moreover, the binding was further validated using the MM/GBSA approach, revealing that these mutations have higher binding energies than the wild-type (WT) NSP13 protein. These findings thus justify the basis of stronger interactions and evasion for these NSP13 mutants. In conclusion, the current findings explored the key features of the NSP13 WT and its mutant complexes, which can be used to design structure-based inhibitors against the SARS-CoV-2 new variants to rescue the host immune system.

## Introduction

Severe acute respiratory syndrome coronavirus 2 (SARS-CoV-2) is the newly emerged virus that causes coronavirus disease 2019 (COVID-19). Its genome contains 5′untranslated region (UTR), 3′UTR, and ORF1a/b that encode 16 non-structural proteins (NSPs), four structural proteins, i.e., spike, envelop, membrane, and nucleocapsid, and nine accessory proteins, which include ORF3a, ORF3b, ORF6, ORF7a, ORF7b, ORF8, ORF9b, ORF9c, and ORF10 (Ribero et al., 2020).

Severe acute respiratory syndrome coronavirus 2 suppresses the immune response of the host during the early phase of infection (Chu et al., 2020). SARS-CoV-2 replicates more efficiently than severe acute respiratory syndrome coronavirus (SARS-CoV) in alveolar macrophages and pneumocytes (both type I and type II) in *ex vivo* lung tissues. Moreover, in the same model, SARS-CoV-2 also suppresses type I/II and III interferons (IFNs). However, more investigations are required on innate immune suppression by SARS-CoV-2, which is associated with viral pathogenesis.

The first line of defense against viral infections, including coronaviruses, is IFN production by the innate immune system. IFN-I production is initiated soon after recognizing pathogen-associated molecular patterns (PAMPs), such as viral mRNA. PAMPs are recognized by retinoic acid-inducible gene 1 (RIG-I) and melanoma differentiation-associated gene 5 (MDA5). When RIG-I and MDA5 are activated, they bind to the CARD domain of mitochondrial antiviral signaling protein (MAVs). When MAVs are activated, they recruit several downstream signaling components to the mitochondria to activate the inhibitors of k-B kinase ε (IKKε) and TANK binding kinase 1 (TBK1), which, in turn, phosphorylate IFN regulatory factor 3 (IRF3) and IRF7. The phosphorylation of IRF3 and IRF7 leads to dimerization and their translocation to the nucleus. Inside the nucleus, they trigger the expression of IFN-1 genes (Sharma et al., 2003; Kawai and Akira, 2007).

The individual protein of SARS-CoV-2 antagonizes IFNβ production by different mechanisms (Lei et al., 2020; Li et al., 2020; Xia et al., 2020; Yuen et al., 2020; Rashid et al., 2021a,b; Vazquez et al., 2021). Among these viral proteins, non-structural protein 13 (NSP13) was found to antagonize IFNβ production by binding to TBK1 to inhibit its phosphorylation and cause decreased activation of IRF3 and IFNβ (Xia et al., 2020; Yuen et al., 2020; Guo et al., 2021; Vazquez et al., 2021).

Severe acute respiratory syndrome coronavirus 2 has undergone various mutations. The mutations produced several variants in different countries. Some of the important variants are called variants of concern (VOCs), including Alpha, Beta, Gamma (P.1), and Delta (B.1.617.2), or variants of interest (VOIs), including Iota (B.1.526), Epsilon (B.1.429/427), Kappa (B.1.617.1), and Mu (Annavajhala et al., 2021; McCallum et al., 2021a,b; Nonaka et al., 2021; Planas et al., 2021). These mutations are responsible for enhanced dissemination, severe clinical outcomes, and increased infectivity of the variants (Chen et al., 2020). These mutations are reported in all viral proteins, including non-structural protein NSP13 (Cao et al., 2021). When compared to the reference strain, Wuhuan-Hu-1 (accession no. NC_045512), the mutations in NSP13 included P77L in Delta (B.1.617.2), Q88H in Iota (B.1.526), D260Y in Epsilon (B.1.429), E341D in Gamma (P.1.1), and M429I in Kappa (B.1.617.1) (Nagy et al., 2021). The mutations P504L and Y541C in NSP13 were important for different disease outcomes (Cao et al., 2021). The function of NSP13 has been reported in several studies to antagonize the production of IFNβ by physically binding to TBK1 and to evade immune response (Lei et al., 2020; Xia et al., 2020; Yuen et al., 2020; Guo et al., 2021; Vazquez et al., 2021). Since various VOIs and VOCs were emerging, we were interested in whether the pathogenesis of these variants might also be contributed by the mutations in NSP13 besides the spike protein in terms of its immune evasion. We were specifically interested in whether these mutations altered the binding efficiencies of NSP13 with TBK1 to antagonize IFNβ production and favor SARS-CoV-2 pathogenesis. In the current study, biophysical and comparative binding approaches were adapted to dissect the roles of NSP13 wild type (WT) and its mutants in the evasion of the immune system through its binding with TBK1. We explored that mutations in NSP13 had higher binding energies compared to the WT. These mutations may therefore justify the basis for stronger interactions with TBK1 and evasion from the immune system.

## Methodology

### Structural Modeling and Validation

A recently reported crystallized structure of NSP13 was retrieved from UniProt (Magrane and UniProt Consortium, 2011) and subjected to structural modeling of SARS-CoV-2 variants using Chimera v_ software (Goddard et al., 2005). Mutations, including P77L, Q88H, D260Y, E341D, and M429I, were introduced to model the mutant structures based on the structure of NSP13 WT. For structural comparison, each mutant was superimposed on the WT, and the root mean square deviation (RMSD) differences were recorded.

### Protein–Protein Docking

Protein–protein docking (NSP13 WT/NSP13 mutants) was used to determine the efficiency of binding of NSP13 with the TBK1 human protein. For this purpose, the HDOCK algorithm was used. The docking interface was visualized by using Guru Interface (Xue et al., 2016).

### Molecular Dynamics Simulations

Dynamic behavior analysis of NSP13 WT and its mutants, P77L, Q88H, D260Y, E341D, and M429I, through molecular dynamics (MD) simulation was determined using AMBER20 (Salomon-Ferrer et al., 2013) using FF14SB force field. For system solvation, an OPC water box was used. For neutralization, Na+ ions were added to the system to balancing it (Price and Brooks, 2004). Energy minimization protocol was used to remove bad clashes from the system, and 3,000 cycles were used for conjugate algorithms (Watowich et al., 1988). For six thousand cycles, the steepest descent algorithm was used (Meza, 2010). The system was equilibrated at 1 atm pressure and heated at 300K. Molecular dynamic simulations were run for 50 ns. Long-range electrostatic integrations, with a cutoff distance of 10.0Å, were treated by the Particle Mesh Ewald algorithm (Salomon-Ferrer et al., 2013). Covalent bonds were treated with the SHAKE algorithm (Kräutler et al., 2001). Amber20 CPPTRAJ package was used to analyze trajectories. Particle Mesh Ewald MD was used to carry out molecular dynamic simulations (Roe and Cheatham, 2013).

### Calculating Binding Free Energy

Actual binding energies were determined for NSP13 WT and NSP13 mutants with TBK1. MM/GBSA approach was used for this purpose, as it is the most acceptable approach for calculating the binding energies of molecular bindings (Khan et al., 2018, 2021b; Ali et al., 2019). GB, SA, vdW, and the electrostatic and free energy of NSP13 WT and NSP13 mutants were calculated (Hou et al., 2011). The equation used to calculate free energy is as follows


(i) "ΔG(bind)=ΔG(complex)-[ΔG(receptor)+ΔG(ligand)]".

(ii) "G=Gbond+Gele+GvdW+Gpol+Gnpol".

Gbond was bond, while Gele was electrostatic. DvdW means van der waals. Gpol was polarly solvated for free energy, and Gnpol was nonpolar solvated free energy. By using the generalized born (GB) solvent methods, the Gnpol and Gpol were analyzed.

## Results

### Mutant Modeling of Non-structural Protein 13 and Its Superimposition on Non-structural Protein 13 Wild-Type

**FIGURE 1:**
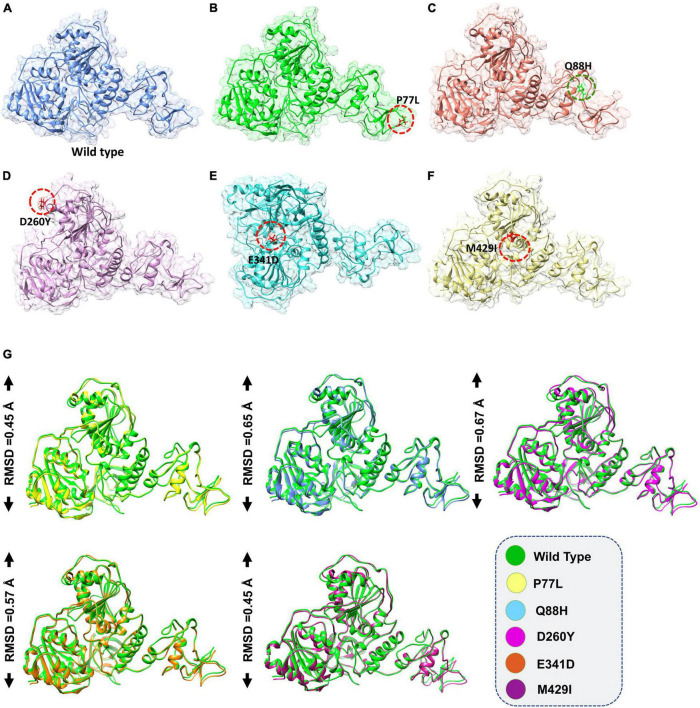
Non-structural protein 13 (NSP13) mutant modeling and the superimposition of NSP 13 wild-type (WT) with NSP 13 mutants. **(A)** NSP13 WT, **(B)** P77L, **(C)** Q88H, **(D)** D260Y, **(E)** E341D, and **(F)** M429I. **(G)** Superimposed structure of NSP13 WT (green) with P77L (yellow), Q88H (cyan), D260Y (light magenta), E341D (orange), and M429I (dark magenta). The root mean square deviation (RMSD) values of each superimposition are shown.

The accession number for the NSP13 WT sequence was NC_045512. To determine whether NSP13 mutants, compared to their WT counterpart, could affect their interaction with TBK1, mutants P77L, Q88H, D260Y, E341D, and M429I were generated by Chimera (Figures 1A–F). The superimposition of these generated mutants followed it on NSP13 WT, and RMSD values were observed. The RMSD differences were substantial for each superimposed structure. Due to mutations in NSP13 WT, the protein conformations and secondary structural elements were altered (Figure 1G). Therefore, it was necessary to analyze the binding efficiency of NSP13 mutants with TBK1. Hence, structural approaches, like biophysical simulations and protein–protein docking, were used to determine the impact of these variations on downstream immune evasion.

### Non-structural Protein 13 Wild-Type-TANK Binding Kinase 1 and Non-structural Protein 13 Mutant-TANK Binding Kinase 1 Docking

The role of NSP13 in immune evasion has been well established. NSP13 is physically bound to TBK1 to inhibit the phosphorylation of TBK1 and IRF3 and hence decrease IFNβ production (Xia et al., 2020). Under this scenario, it was indispensable to perform a binding analysis of NSP13 WT and NSP13 mutants with TBK1, and the proteins inside cells interact to regulate many biological processes (Khan et al., 2021a; Rashid et al., 2021b). The binding energies and structural determinants of these interactions are important steps to study the regulations of these processes. Binding affinity, which regulates molecular interactions, determines if the formation of complexes occurs under definite conditions (Smith and Sternberg, 2002). Protein–protein dockings of NSP13 WT and its various mutants were performed using HDOCK so that the level of pathogenicity of different mutants of SARS-CoV-2 could be determined.

**FIGURE 2:**
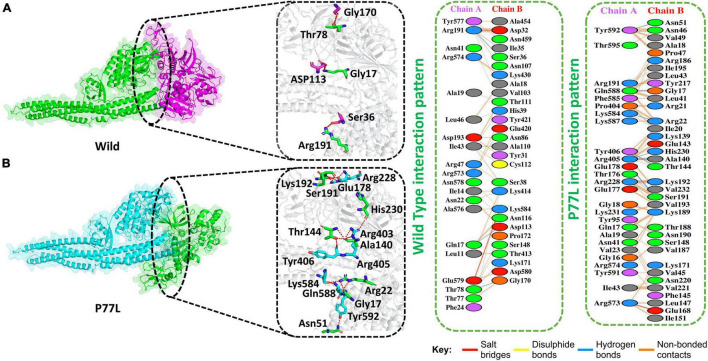
Non-structural protein 13 WT and P77L mutant complex docking. **(A)** NSP13 WT and TANK binding kinase 1 (TBK1) key hydrogen bonding interactions with stick representations (left). Hydrogen bonds, non-bonded contacts, and salt bridges are shown by a 2D interaction (right). **(B)** P77L mutant and TBK1 key hydrogen bonding interactions along with their stick representation (left). Hydrogen bonds, non-bonded contacts, and salt bridges are shown by a 2D interaction (right).

The docking score predicted by HDOCK online server was −175 kcal/mol for the TBK1-NSP13 WT complex, and the PDBsum analysis showed that these structures formed three hydrogen bonds, two salt bridges, and 156 non-bonded contacts. The hydrogen bonds between TBK1-NSP13 WT included Arg191–Ser36, Gln17–Asp113, and Thr78–Gly170. However, the residues Arg191–Asp32 and Glu579–Lys584 formed salt bridges (Figure 2A).

Delta variant B.1.617.2 was first identified in India in December 2020 and spread to other countries. This variant harbor 10 mutations in spike protein. Since the NSP13 protein was responsible for antagonizing IFNβ production, we explored NSP13 mutations in the Delta variant. Indeed at amino acid position 77, NSP13 was mutated, i.e., P77L. Therefore, we were interested if this mutation could enhance the function of NSP13 in immune evasion and might help delta variants become more pathogenic. We, therefore, determined the docking score for this mutation and compared it to its WT counterpart.

The HDOCK docking score for P77L (TBK1-P77L) was −204 kcal/mol, the substitution of amino acid highly increased the interaction of NSP13 and TBK1, and 10 hydrogen bonds, one salt bridge, and 263 non-bonded contacts were formed (Figure 2B). The key residues to form hydrogen bonds were Tyr592–Asn51, Gln588–Gly17, Lys584–Arg22, Tyr406–Thr144, Arg405–Ala140, Arg405–His230, Glu178–Ser191, and Arg228–Lys192. Residues Arg573–Glu168 formed salt bridges. The P77L mutation was found to increase the function of the NSP13 protein by binding to TBK1 more strongly compared to WT. Therefore, it could be deduced that P77L might suppress IFNß production more stringently and make this virus more pathogenic.

**FIGURE 3:**
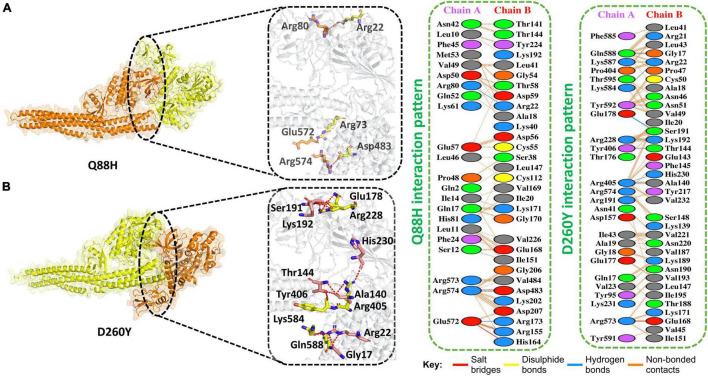
Q88H and D260Y mutant complex docking. **(A)** Q88H mutant and TBK1 key hydrogen bonding interactions and representations of bonds by sticks (left). Hydrogen bonds, non-bonded contacts, and salt bridges are shown by a 2D interaction (right). **(B)** D260Y mutant and TBK1 key hydrogen bonding interactions and representations of bonds by sticks (left). Hydrogen bonds, non-bonded contacts, and salt bridges are shown by a 2D interaction (right).

The Iota variant (B.1.526) was first detected in the United States of America in November 2020. It has seven mutations in spike protein. Moreover, its NSP13 protein also harbored mutation at amino acid 88, i.e., Q88H. We, therefore, determined if this mutation could enhance the binding of NSP13 with TBK1 compared to WT. We first determined the docking score for this mutation and found that the HDOCK docking score for Q88H (TBK1-Q88H) was −210 kcal/mol. This analysis revealed that three salt bridges and three H-bonds were present. In addition, 210 non-bonded contacts were observed for this mutation. The Q88H mutation was observed to bind more stringently to TBK1 compared to WT. Residues Arg574–Asp483, Glu572–Arg155, and Glu572–His164 formed the salt bridges. Hydrogen bond-forming residues were Arg80–Arg22, Arg574–Asp483, and Glu572–Arg173 (Figure 3A).

In addition, the Epsilon variant (B.1.427/B.1.429) emerged in the United States in June 2020. This variant also has a mutation in NSP13 at position 260, i.e., D260Y. Therefore, we determined the docking score for this mutation and found that the HDOCK docking score for D260Y (TBK1-D260Y) was observed to be −227 kcal/mol. This analysis revealed that one salt bridge and eight H-bonds were present. In addition, 277 non-bonded contacts were observed for this mutation. D260Y mutation enhanced the binding with TBK1. The salt bridge was formed between Arg573-Glu168. The hydrogen bonds were formed between these residues Gln588–Gly17, Lys584–Arg22, Glu178–Ser191, Arg228–Lys192, Tyr406–Thr144, Arg405–His230, and Arg405–Ala140 (Figure 3B).

**FIGURE 4:**
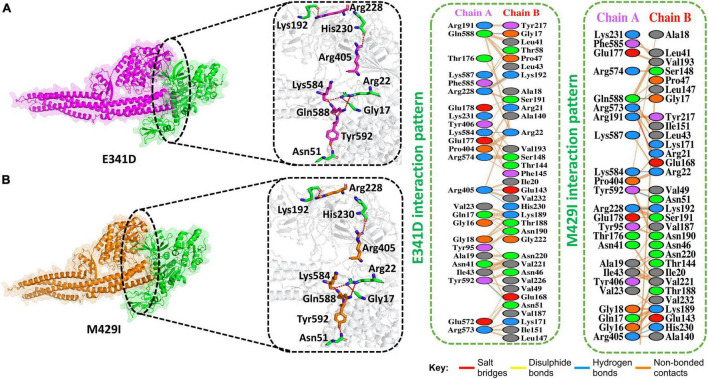
E341D and M429I mutant complex docking. **(A)** E341D mutant and TBK1 key hydrogen bonding interactions and stick representations of bonds (left). Salt bridges, hydrogen bonding, and non-bonded interactions are shown by a 2D interaction (right). **(B)** M4291 mutant and TBK1 key hydrogen bonding interactions and stick representations of bonds (left). Salt bridges, hydrogen bonding, and non-bonded interactions are shown by a 2D interaction (right).

Gamma (P.1) was first identified in Brazil in December 2020. This variant also has a mutation in its NSP13 at amino acid position 341, i.e., E341D. We determined the docking score for this mutation and found that the predicted score of HDOCK for E341D (TBK1-E341D) was −245 kcal/mol. This analysis revealed the presence of five hydrogen bonds. In addition, 260 non-bonded contacts were observed for this mutation. Gln588–Gly17, Arg228–Lys192, Lys584–Arg22, Arg405–His230, and Tyr592–Asn51 formed hydrogen bonds between TBK1 and E341D mutant (Figure 4A).

The Kappa (B.1.617.1) variant was detected in India in December 2020. This variant also has a mutation in its NSP13 at amino acid position 429, i.e., M429I. We determined the docking score for this mutation and found that the HDOCK docking score for M429I (TBK1-M429I) was −229 kcal/M. The replaced amino acids increased the interactions of NSP13 to TBK1 compared to the WT complex. Five hydrogen bonds, one salt bridge, and 215 non-bonded contacts were reported (Figure 4B). Gln588–Gly17, Lys584–Arg22, Tyr592–Asn51, Arg228–Lys192, and Arg405–His230 residues formed the hydrogen bonds. Arg573–Glu168 amino acids participated in the salt bridge formation.

### Structural–Dynamic Features of Non-structural Protein 13 Wild-Type and Its Mutant Complexes

**FIGURE 5:**
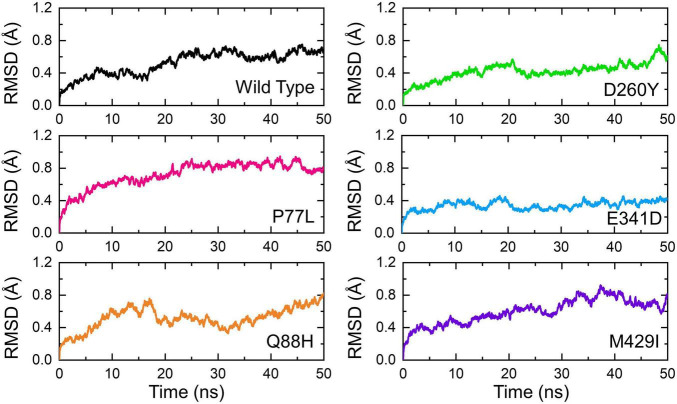
Dynamic stability of the NSP13 WT and mutant complexes calculated as RMSD over the simulation time in nanoseconds. The RMSD of NSP13 WT is shown in black. For the mutants, the RMSDs are shown in different colors. RMSDs, root mean square deviations.

Understanding the dynamic features of protein–protein complexes provides a better view of the regulation of different molecular mechanisms of the cell in a coordinated manner. The context of protein–protein interaction in the interpretation of host–pathogen interaction always aids in the comprehension of transmission and mechanism of the pathogenesis of the invader. Many previous studies used the dynamics of molecular machinery to demonstrate the exact mechanism of pathogenesis. In this regard, exploring the structural and dynamic stability of biological macromolecular association provides an understanding of the binding affinity. By employing the dynamic stability equation herein, we estimated the structural stability of each complex as RMSD (Figure 5). During the 50-ns simulation, the RMSD of the WT gradually increased over time. The average RMSD observed was 0.3Å during the first 10 ns; however, the RMSD increased onward. During the last 35 ns, the RMSD value then increased further and reached 0.5Å. Minor structural deviations at 22, 28, 35–40, and 46 ns were reported.

On the other hand, the P77L mutant also exhibits a similar pattern of RMSD to the WT. The RMSD increased gradually over the simulation time; however, the structural deviation at different time intervals was minimal. The average root mean square fluctuation (RMSF) was reported to be 0.6Å. Unlike the WT and P77L, the Q88H complex demonstrated a higher structural instability. The RMSD gradually increased between 0 and 10 ns and then abruptly decreased for a short interval between 11 and 15 ns. More structural deviation was observed until 50 ns.

In contrast, D260Y and E341D displayed a more stable dynamic trend than the other complexes. Except for a minor deviation between 20–22 and 46–50 ns, the overall dynamics of the D260Y mutant revealed a very stable behavior, with an average RMSD of 0.3Å. Similarly, with few structural deviations between 15–20, 32–40, and 41–42 ns, the mean RMSD was also reported to be 0.3Å for E341D. Furthermore, the M429I mutant demonstrated similar deviations as the WT, P77L, and Q88H mutant. The average RMSD for M429I was 0.65Å; however, an increasing trend was reported – for instance, a previous study reported that mutations with increasing instability produce the function of radical protein, implying that these mutations have evolved with radical function and have increased infectivity (Hussain et al., 2020).

**FIGURE 6:**
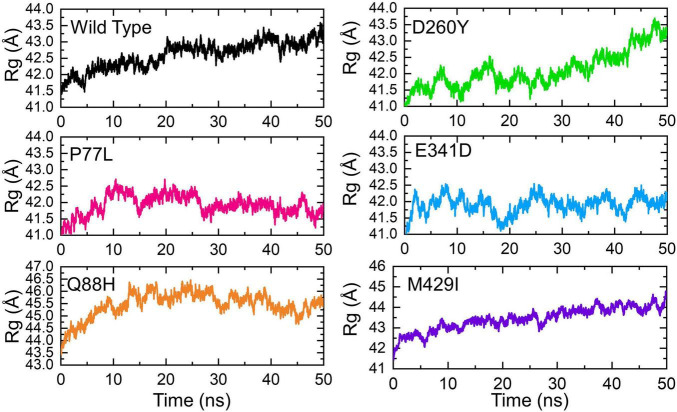
Structural compactness of the NSP13 WT and mutant complexes calculated as radius of gyration (Rg) over the simulation time in nanoseconds. The Rg of WT is represented in black, while different colors are used for the mutants. Rg, radius of gyration.

Next, we calculated the structural compactness of each complex as the radius of gyration (Rg) over the simulation time in nanoseconds. Structural compactness, in a dynamic environment, showed that binding and unbinding events of the interacting partners occurred during the simulation. Herein the Rg of each complex was calculated and shown (Figure 6). The WT gradually lost the compactness, and the Rg value increased with time. The average Rg value for the WT was recorded to be 42.5Å. The compactness of P77L was different from the WT. The structural compactness initially decreased during the first 10 ns; however, the Rg value then became flat. The Rg fluctuated between 41.5 and 42.0Å. The Rg increased between 15 and 20 ns and then decreased and remained persistent until 50 ns. The average value for the Q88H was reported to be higher; however, the Rg remained unchanged after 10 ns. The D260Y and M429I demonstrated a similar behavior. The Rg gradually increased, and many fluctuations were observed. The E341D mutant reported lower Rg with continuous increase or decrease in the Rg value during the simulation period. Consequently, this analysis showed that mutations had passed significant binding and unbinding events, which caused structural perturbations at different time intervals. Similar binding and unbinding events for other variants have previously been reported (Khan et al., 2021b).

**FIGURE 7:**
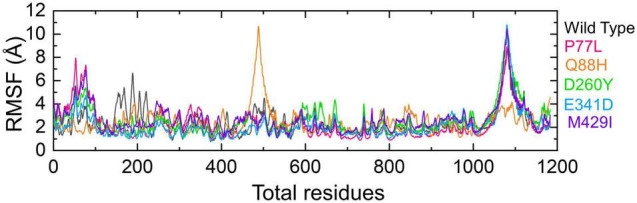
Residual flexibility of the NSP13 WT and mutant complexes calculated as root mean square fluctuation (RMSF). The RMSFs of WT are represented in black, while different colors are used for mutants. RMSF, root mean square fluctuation.

The RMSF of residues was calculated to estimate the residual flexibility. Understanding the residual flexibility of the system is key to highlighting residues vital in holding the interacting ligand and overall stabilization of the complex. The residual flexibility demonstrated variations in different regions, i.e., 10–100 and 1,000–1,150, for all the complexes except Q88H at the position 1,000–1,150. The WT demonstrated different flexibility patterns, in particular in the region between 150 and 200. Similarly, the Q88H exhibited more frequent fluctuations between 480 and 550. Moreover, no significant fluctuation was observed for other mutants except in the region of 1,400–1,500. These results confirmed that the difference in the dynamic flexibility resulted in variable conformational optimization and binding of the interacting proteins as indicated by the RMSF of each complex (Figure 7).

**FIGURE 8:**
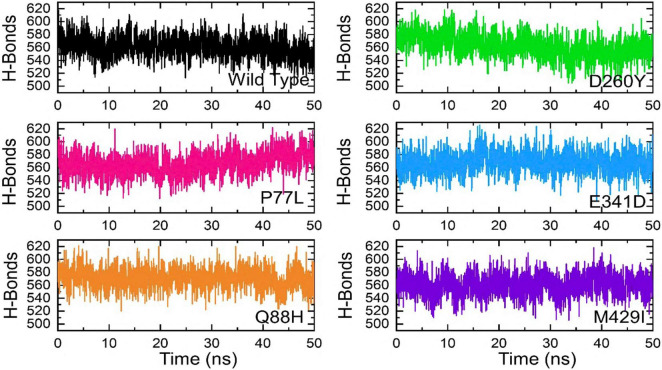
Hydrogen bonding of the WT and mutant complexes calculated as a function of time. The H-bonds for WT are shown in black, while for mutants these are shown in different colors.

The binding variations were further explored through hydrogen bonding analysis as a function of time. The total number of hydrogen bonds was calculated, including the inter- and intra-molecules. The results from 2,500 structural frames revealed that the average number of hydrogen bonds in WT was 554, 583 in P77L, 579 in Q88H, 565 in D260Y, 585 in E341D, and 578 in M429I complex, respectively. The results indicated that significant hydrogen bonding reprogramming took place. The increased bonds in the mutant complexes delineated stronger affinity toward the interacting receptor, which resulted in higher affinity. The hydrogen bond graphs are shown (Figure 8).

The strength of a biomolecular association can be determined by estimating the binding affinity of the two interacting macromolecules. Computation of binding free energy using MM/GBSA methods is the most commonly used approach to re-rank docking conformations *via* calculations of structural–dynamic stability, the strength of interacting key hotspots, and total binding energies. The method mentioned above is computationally inexpensive than any other method, i.e., the alchemical free energy calculation method. The MM/GBSA technique is considered more accurate and comprehensive than the conventional scoring functions. Thus, to re-evaluate the binding scores of the WT and mutant complexes, we employed MM/GBSA approach using 2,500 structural frames (Table 1). The binding energies for NSP13 WT and mutant P77L, Q88H, D260Y, E341D, and M429I were −74.72, −84.64, −91.36, −90.80, −85.17, and −81.98 kcal/mol, respectively. The electrostatic interactions mainly guide the higher binding affinity. In conclusion, our findings showed that the mutant complex bound more robustly than WT, thus contributing toward higher infectivity and immune evasion.

**TABLE 1:** MM/GBSA analysis of the wild-type (WT) and mutant complexes.

Complex	vdW	ELE	GB	SA	Total
WT	−139.00	−280.54	361.48	−16.66	−74.72
P77L	−190.54	−211.66	340.29	−22.73	−84.64
Q88H	−152.51	−263.89	343.95	−18.91	−91.36
D260Y	−194.07	−218.37	345.16	−23.52	−90.80
E341D	−192.69	−243.29	374.08	−23.27	−85.17
M429I	−185.02	−199.99	325.25	−22.22	−81.98

*vdW, van der waal; ele, electrostatic; GB, polar solvation; and SA, surface area. All the energies are given in kcal/mol.*

## Discussion

The SARS-COV-2 NSP13 protein is well known for its ability to evade the host immune system by binding to TBK1 and inhibiting the phosphorylation of TBK1 and IRF3 from suppressing IFNβ production and signaling. Since several mutations have been observed in NSP13 (Xia et al., 2020; Yuen et al., 2020; Guo et al., 2021; Vazquez et al., 2021), it was necessary to elucidate the effects of different NSP13 mutants on binding with TBK1. Using computational tools, understanding the impact of different mutations is an accurate and traditional approach (Hussain et al., 2020). Computational modeling and simulation approaches were employed in the current study to dissect the binding of NSP13 mutants with TBK1.

The binding energies and structural determinants of these interactions are important steps to study regulations of protein–protein interactions. Dockings for protein–protein of NSP13 WT and its various mutants were performed using HDOCK. These docking results suggested that the scores for these mutants increased compared to those of WT, suggesting that the binding affinity with TBK1 may increase (Figures 2–4). An interaction analysis revealed that the number of hydrogen bonds and salt bridges (except E341D) also increased in these mutants compared to those in WT (Figures 2–4). Our results indicated that these mutants might favor a more efficient evasion of the host immune system than WT.

The structural instabilities from minor to large, as shown by RMSD values, were observed (Figure 5). However, mutations with increasing instability produced the function of the radical protein, thus implying that these mutations have evolved with radical function and may increase their infectivity (Hussain et al., 2020). Our findings revealed that the WT exhibited a different bonding network and dynamics than the mutants mentioned above. These mutations produced radical function by readjusting the protein topology. Previous mutations with radical functions and decreased stability were also reported to impact the binding and molecular function of the interacting molecules (Wang et al., 2021). These findings also revealed a substantial reprogramming of hydrogen and other bonds, consequently increasing the binding affinity. In addition, these findings were validated by estimating the binding free energy. It was observed that the mutations, in particular Q88H, have a higher binding affinity than WT and even other mutants (Table 1).

Similarly, the Q88H exhibited the least fluctuation among all the complexes, except in the region between 480 and 550 (Figure 7). However, experimental validations are required for these analyses in the future. It has been shown that NSP13 interacts with TBK1 and inhibits the phosphorylation of IRF3 and TBK1 (Xia et al., 2020). Therefore, it would be interesting to determine the binding of NSP13 mutants experimentally with TBK1 and whether the mutants bind more stringently to TBK1 than WT. Moreover, the differential effects on phosphorylation levels of TBK1 and IRF3 and inhibition of IFNβ production by NSP13 WT and its various mutants could be determined experimentally. Our biochemical data could infer that, compared to WT, various mutants of NSP13 may show more inhibitory effects on IFNβ production; however, experimental validations are indispensable. A mutational study of SARS-CoV-2 variants has revealed the basis for tighter binding and its correlation with infectivity. Moreover, immune evasion has also been revealed through extensive interaction and dynamics studies (Khan et al., 2021a). Therefore, continuous mutational analysis is highly required to determine the disease status and outcomes.

## Data Availability Statement

The raw data supporting the conclusions of this article will be made available by the authors, without undue reservation.

## Author Contributions

ST designed the project and edited the manuscript. FR did some analysis and wrote the manuscript. MS and AS did the biophysical and structural analysis. ED, HW, and SC edited the final manuscript. All authors contributed to the article and approved the submitted version.

## Conflict of Interest

The authors declare that the research was conducted in the absence of any commercial or financial relationships that could be construed as a potential conflict of interest.

## Publisher’s Note

All claims expressed in this article are solely those of the authors and do not necessarily represent those of their affiliated organizations, or those of the publisher, the editors and the reviewers. Any product that may be evaluated in this article, or claim that may be made by its manufacturer, is not guaranteed or endorsed by the publisher.
